# Culture, sex and social context influence brain-to-brain synchrony: an fNIRS hyperscanning study

**DOI:** 10.1186/s40359-024-01841-3

**Published:** 2024-06-14

**Authors:** Mengyu Lim, Alessandro Carollo, Andrea Bizzego, Annabel SH Chen, Gianluca Esposito

**Affiliations:** 1 Psychology Program, School of Social Sciences, Nanyang Technological University, Singapore, Singapore; 2 Department of Psychology and Cognitive Science, University of Trento, Rovereto, Italy

**Keywords:** Culture, Sex, Personality, fNIRS, Interpersonal synchrony, Role-play, Empathy, Big five inventory, Interpersonal reactivity index, Prefrontal cortex

## Abstract

**Background:**

Unique interpersonal synchrony occurs during every social interaction, and is shaped by characteristics of participating individuals in these social contexts. Additionally, depending on context demands, interpersonal synchrony is also altered. The study therefore aims to investigate culture, sex, and social context effects simultaneously in a novel role-play paradigm. Additionally, the effect of personality traits on synchrony was investigated across cultures, and a further exploratory analysis on the effects of these variables on pre- and post-session empathy changes was conducted.

**Methods:**

83 dyads were recruited in two waves from Singapore and Italy and took part in a within-subjects session where they interacted with each other as themselves (Naturalistic Conversation) and as others (Role-Play and Role Reversal). Big Five Inventory (administered pre-session) and Interpersonal Reactivity Index (administered pre- and post-session) were used as measures of personality and empathy respectively, while synchrony was measured using hyperscanning functional near-infrared spectroscopy in the prefrontal cortex. After data-preprocessing and preliminary analyses, a mixture of multiple linear regression and exploratory forward stepwise regression models were used to address the above study aims.

**Results:**

Results revealed significant main and interaction effects of culture, sex and social context on brain-to-brain synchrony, particularly in the medial left cluster of the prefrontal cortex, and a unique contribution of extraversion and openness to experience to synchrony in the Italian cohort only. Finally, culture-driven differences in empathy changes were identified, where significant increases in empathy across sessions were generally only observed within the Singaporean cohort.

**Conclusions:**

Main findings indicate lowered brain-to-brain synchrony during role-playing activities that is moderated by the dyad’s sex make-up and culture, implying differential processing of social interactions that is also influenced by individuals’ background factors. Findings align with current literature that role-playing is a cognitively demanding activity requiring greater levels of self-regulation and suppression of self-related cognition as opposed to interpersonal co-regulation characterized by synchrony. However, the current pattern of results would be better supported by future studies investigating multimodal synchronies and corroboration.

## Background

Social interactions require the involvement of at least two individuals in a dynamic, bidirectional exchange. During the exchange, individuals attempt to coordinate with each other, both verbally and nonverbally, resulting in a natural attunement, or co-regulation, of all parties involved. Social scientists term this phenomenon “synchrony”, which characterizes the unique dynamic arising from the presence and interactions of multiple parties in a social exchange. Synchrony can be observed at various layers of the interaction, ranging from verbal [1] and behavioral [2] markers, to neurological [3] and physiological signals [4].

Social interactions are never conducted in a vacuum; participating individuals bring with them their sociocultural backgrounds, personal histories and unique perspectives on themselves and the world around them. This study of individual characteristics has found that behaviors can be altered systematically along several lines including culture and sex. The most popular dimension of individualism-collectivism in characterizing culture [5] reveals differences in group behavior depending on the prevailing cultural background of the group, as well as the cultural identification of the individual [6]. Collectivistic group behavior predicts greater extents of cooperation [7] and appears to be driven by a stronger sense of group loyalty [8] and increased sensitivity to social cues [6]. Sex differences (in this paper, the focus is only on biological males and females) have also been found to contribute to differences in human behavior, particularly in social settings. For example, approaches to social situations, affect expression [9] and preference for social interactions [10] have all been found to be predicted by individual sex and appear to be driven by a combination of biological differences and socialization [11]. Furthermore, these broad categories have been found to influence each other to form culture and sex interactions [12], paving the way forward for more nuanced simultaneous investigations of multiple constructs.

Synchrony observed in social interactions has therefore been found to be moderated by the above factors related to individual differences, including the culture and sex of those involved in the interaction. For example, behavioral studies have found significant differences in frequency of head motion synchrony between Japanese and English participants [13], as well as in the expression of joint emotion between Indian and American participants [14]. In these studies, it appears that culture is a driving factor in the expression and interpretation of behavior which influences the degree of synchrony between individuals. For example, nodding (i.e., head motion) is interpreted more positively in Eastern rather than Western nations [15, 16]. Similarly, synchrony has also been found to differ based on participant sex make-up when considering neurological levels of interpersonal coordination, where females are generally more likely to display greater brain-to-brain synchrony [17, 18]. A survey of the above studies investigating the effect of these individual factors on interpersonal synchrony suggests that these factors are intrinsically related to the quality of observed synchrony during social interactions. However, literature remains inconsistent on the size and overall direction of relationships between culture and sex on interpersonal synchrony. For example, other studies have uncovered no significant differences between culture (e.g., between American/English and Japanese participants [19]), instead identifying other salient moderators of synchrony in terms of situational demand. Likewise, there are also conflicting results on the effect of sex on synchrony, with Tschacher and colleagues [18] observing more non-verbal synchrony among male-male dyads instead. It should be noted that while most of the studies of sex and culture factors in synchrony rely on behavioral measures, the use of neuroimaging tools in synchrony research is a burgeoning field worth further investigation. Commonly, the simultaneous recording using electroencephalogram and functional near-infrared spectroscopy (fNIRS) tools, known as hyperscanning, is used to visualize patterns of brain activation that are common across individuals as they partake in the same activity together [20–22].

Another salient research gap lies in the situational context of the social interaction, as hinted by Fujiwara and colleagues [19]. Depending on the demands of the context, synchrony exhibited by the same interacting individuals will differ. For example, cooperative as compared to competitive interactions elicit greater brain-to-brain and behavioral synchrony [23, 24], while negative perception of an interaction partner reduced behavioral synchrony [25]. A particular social context that had not yet been investigated in detail despite its prevalence is during role-playing activities. Role-play is a common activity implemented in teaching [26–28], entertainment [29–31] and clinical [32, 33] contexts, used to create immersive experiences that convey alternative perspectives directly to the role-player. Commonly, role-playing is related to positive learning and personal outcomes, most noteworthy of which is an increase in empathy due to more effective perspective taking [34, 35], as well as the mirroring of said persona’s typical behaviors [36]. This may be attributable to a unique phenomenon that occurs during role-play unlike other social interactions: during role-play, all involved parties suspend their social identities in the real world in favor of portraying another persona in a shared hypothetical scenario [37, 38]. This raises interesting questions about how role-playing will influence the innate interpersonal synchrony displayed by participating individuals. The portrayal of other characters may prove to disrupt interpersonal synchrony otherwise seen during a typical interaction. Additionally, the individual factors described above (culture, sex) have already been found to also have an effect on observed role-play behavior. Gosso and colleagues [39], Haight and colleagues [40] and Edwards [41], through observing role-play among children, suggest that the opportunity to engage in role-play and the content of these interactions vary across cultures. A cross-cultural comparison between American and Italian participants during role-play also showed a greater likelihood of affect expression among Italians [42], which holds implications when considering that shared affect is also a key indicator of interpersonal synchrony [14]. Likewise, females are more likely to report greater dedication to role-playing activities as compared to males [37], with some preliminary findings pointing to sex differences in language use when role-playing [43]. Taken together, while no study has examined the relationship between role-play and synchrony, as well as the individual factors influencing this relationship, related literature studying cultural and sex influences on role-play and synchrony suggest that these variables are correlated with each other. Interpersonal synchrony may be predicted not only by the social context (i.e., role-playing technique), but also by culture and sex of the participating individuals.

Finally, the variation in personalities across individuals cannot be disregarded. It was previously found that individuals with higher openness to experience [18], agreeableness [44] and extraversion [45] are more likely to display synchrony, perhaps pointing to a greater likelihood of attending and responding to other individuals’ cues during the interaction. In all the above studies on personality and synchrony, same-sex dyads composed of strangers were paired up and tasked to undergo social interactions. In Tschacher and colleagues’ [46] study, it was found that male dyads and dyads high in Openness to Experience demonstrated longer instances of non-verbal synchrony. Arellano-Véliz and colleagues’ [45] study found a significant effect of Extraversion increasing interpersonal synchrony among dyads who rated similarly in this trait (as opposed to participants who rated dissimilarly in Extraversion). While these studies measured behavioral aspects of synchrony, Zhang and colleagues’ [44] study made use of fNIRS to observe synchrony at the neurological level. In their study, it was revealed that Extraversion and Agreeableness were related to higher brain-to-brain synchrony and greater levels of cooperation among participants [44]. In parallel, studies investigating personality and role-play also point to the relationship between openness to experience and immersive behaviors during role-play [47, 48], perhaps acting as a predisposing factor to pursue role-playing activities [49]. However, the effect of personality profiles when considering interpersonal synchrony during role-play is yet to be elucidated.

### The present study

As synthesized above, the current literature has several gaps that will be addressed in the present study: firstly, the simultaneous investigation of culture and sex and their relationships to interpersonal synchrony, particularly during different social contexts (i.e., role-play). This is the main objective of the study, and the present study will make use of cross-cultural data collection (in Singapore and Italy) with both male and female participants in a within-subjects research design to address this gap. The subsequent objectives are exploratory in nature: An additional layer of nuance is provided in the present study through an exploratory analysis of participants’ personality profiles and their effect on observed interpersonal synchrony across cultural cohorts. Finally, to expand upon this line of inquiry with potential application to outcomes commonly observed as a result of role-play, this study also incorporates empathy measures to determine if culture, sex, personality and role-play techniques have an effect on participants’ reported changes in empathy. Empathy was chosen as a key construct for exploration in the final research objective because of the strong relationship between role-play and empathy [35, 50–52], particularly due to perspective taking and reappraisal [53, 54] processes that occur during these activities that have the potential to enhance one’s understanding and empathy towards a targeted other. Additionally, empathy has also been found to be influenced by culture [55–57] and sex factors [58]. On an interpersonal level, numerous studies have identified that the dyad’s empathy levels have an effect on the extent of observed synchrony [59, 60], and the opposite relationship (i.e., synchrony has a positive effect on the dyad’s empathy for each other) has likewise been reported [61–63]. The theoretical framework offered in Tzanaki [59] proposes a dual feedback loop between empathy and interpersonal synchrony where one can enhance the other in a social interaction.

To address these gaps, the study measures interpersonal synchrony in terms of brain-to-brain synchrony with fNIRS, and implements a within-subjects experimental paradigm where participating dyads go through a series of typical and role-played interactions. Research questions are formulated as follows:

1. Do social contexts (i.e., experimental conditions), dyadic sex, and cultural cohort affect brain-to-brain synchrony during social interactions?
2. How do dyadic personality traits contribute to brain-to-brain synchrony across cohorts?
3. Which of the above factors influence change in pre- and post-session empathy?

Due to conflicting reports in literature and the pioneering nature of this study in examining interpersonal synchrony during role-play, no hypotheses were put forth. Additionally, it should be noted that a majority of prevalent literature operationalise interpersonal synchrony in terms of behavioral measures. It remains unclear if synchrony along various modalities are equivalent in strength and direction, and this issue is further discussed in the Limitations subsection below. Nonetheless, the present study focuses on brain-to-brain synchrony, and specifically synchrony in the prefrontal cortex due to its unique contribution to social cognition and higher level executive functions that is related not only to role-play [64–66], but also more generally to perspective taking and empathy [67–69]. Past studies investigating synchrony in social interactions have also measured prefrontal cortical activity using fNIRS hyperscanning [44, 70–73].

## Methods

The study’s research design, experimental conditions and session procedures have been previously published in Lim and colleagues [64] investigating individual brain activation rather than interpersonal synchrony.

### Participants

Data was collected in two waves, with the first being in Singapore (*N* = 82; 41 dyads) from 2021 to 2022, and the second being in Italy (*N* = 84; 42 dyads) from 2022 to 2023. Singapore is a nation in Southeast Asia with majority ethnic Chinese and is generally representative of a collectivistic culture [74], while Italy is a nation in Western Europe and is generally representative of an individualistic culture [75]. Demographic details of both cohorts are summarized in Table 1. All participants are existing friends with each other sharing a peer relationship and are healthy adults (i.e., no diagnosed medical or psychological conditions) aged from 18 to 35. Participants were recruited via university networks, as well as through word-of-mouth and social media. The study’s procedure and materials are common across both cohorts, and approved by the Ethics Committees of both Nanyang Technological University (IRB 2021-03-013) and University of Trento (2022-059).

**Table 1:** Participant demographic information

Demographic	Singapore	Italy
Dyad sex	24 female-female	25 female-female
	11 male-male	17 male-male
	6 female-male	
Age	21.95, SD = 3.11	23.36, SD = 2.59

### Equipment and materials

#### Functional near-infrared spectroscopy

**Fig. 1:**
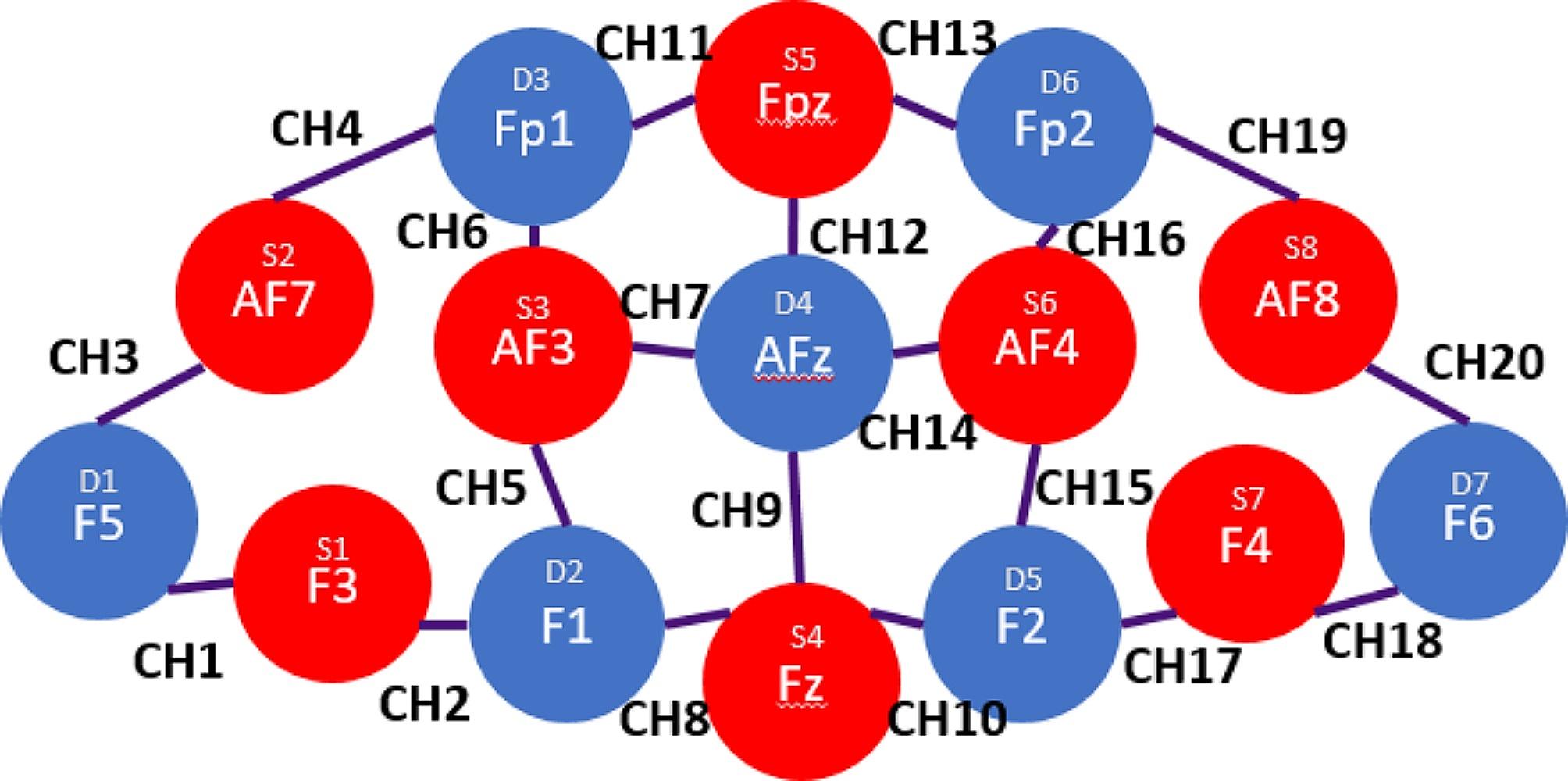
fNIRS prefrontal cortex configuration and its corresponding international 10–20 EEG position. .*Note*: Purple lines indicate approximate areas where channels are formed

Hyperscanning fNIRS (NIRSport and NIRSport2, NIRx Medical Technologies LLC) were used to measure brain activity in participants’ prefrontal cortices during the study. For the Singapore cohort, the NIRStar software (v15.2, Windows 64-bit; compatible with NIRSport) was used for data acquisition on a pre-built 8 × 7 channel configuration of the prefrontal cortex, while the Italy cohort used the Aurora fNIRS software (Windows 64-bit; compatible with NIRSport2) of the same configuration. This configuration is analogous to the international 10–20 electroencephalogram (EEG) system [76], forming a total of 20 fNIRS channels using 8 sources and 7 detectors (Fig. 1). Using AtlasViewer (v2.44.0, Windows 64-bit; [77]), the corresponding Montreal Neurological Institute (MNI) coordinates of the optodes in a standard configuration are reported in Table 2. The NIRSport has a programmed sampling rate of 7.81 Hz and uses near-infrared wavelengths that are 760 nm and 850 nm long [78], while the NIRSport2 has a sampling rate of 10.17 Hz.

**Table 2:** Approximate MNI coordinates

Channel	Source/10–20 position (x, y,z)	Detector/10–20 position (x, y,z)
1	1/F3 (-50.6, 24.5, 25.0)	1/F5 (-64.8, 46.3, 15.8)
2	1/F3 (-50.6, 24.5, 25.0)	2/F1 (-28.6, 14.6, 34.3)
3	2/AF7 (-52.4, 74.2, 23.9)	1/F5 (-64.8, 46.3, 15.8)
4	2/AF7 (-52.4, 74.2, 23.9)	3/Fp1 (-30.5, 80.8, 36.7)
5	3/AF3 (-44.9, 51.3, 37.1)	2/F1 (-28.6, 14.6, 34.3)
6	3/AF3 (-44.9, 51.3, 37.1)	3/Fp1 (-30.5, 80.8, 36.7)
7	3/AF3 (-44.9, 51.3, 37.1)	4/AFz (-3.5, 43.2, 46.1)
8	4/Fz (-3.0, 13.0, 40.1)	2/F1 (-28.6, 14.6, 34.3)
9	4/Fz (-3.0, 13.0, 40.1)	4/AFz (-3.5, 43.2, 46.1)
10	4/Fz (-3.0, 13.0, 40.1)	5/F2 (25.8, 18.2, 35.4)
11	5/Fpz (-5.1, 81.5, 40.0)	3/Fp1 (-30.5, 80.8, 36.7)
12	5/Fpz (-5.1, 81.5, 40.0)	4/AFz (-3.5, 43.2, 46.1)
13	5/Fpz (-5.1, 81.5, 40.0)	6/Fp2 (23.0, 80.9, 38.1)
14	6/AF4 (40.6, 55.8, 36.1)	4/AFz (-3.5, 43.2, 46.1)
15	6/AF4 (40.6, 55.8, 36.1)	5/F2 (25.8, 18.2, 35.4)
16	6/AF4 (40.6, 55.8, 36.1)	6/Fp2 (23.0, 80.9, 38.1)
17	7/F4 (48.3, 26.4, 23.9)	5/F2 (25.8, 18.2, 35.4)
18	7/F4 (48.3, 26.4, 23.9)	7/F6 (61.2, 54.2, 12.0)
19	8/AF8 (47.5, 84.2, 18.9)	6/Fp2 (23.0, 80.9, 38.1)
20	8/AF8 (47.5, 84.2, 18.9)	7/F6 (61.2, 54.2, 12.0)

#### Big five inventory

The English [79] and validated Italian versions [80] of the BFI were used for Singapore and Italy cohorts respectively. The BFI is a measure of five personality traits with 44 items rated on a 5-point Likert scale. The BFI is interpreted based on five dimensions of personality, namely Openness to experience (versus Closedness to experience), Conscientiousness (versus Lack of direction), Extraversion (versus Introversion), Agreeableness (versus Antagonism) and Neuroticism (versus Emotional stability). Based on extant literature, the BFI shows good reliability and validity [80–84].

#### Interpersonal reactivity index

The English [85] and validated Italian versions [86] of the IRI were used for Singapore and Italy cohorts respectively. The IRI is a measure of empathy with 28 items rated on a 5-point Likert scale. The IRI may be interpreted as a global score, as well as its constituent subscales: Fantasy, Empathic Concern, Perspective Taking, and Personal Distress. In the present study, both approaches are taken during subsequent analysis. Additionally, to more accurately capture the change in empathy felt towards the participants’ role-playing partners, the items were adapted following Péloquin and Lafontaine [87] to refer to a specific individual (i.e., their partner) where applicable in the present study. Based on extant literature, the IRI shows good reliability and validity [85, 87].

### Procedure

**Fig. 2:**
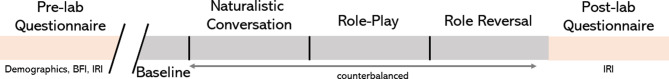
Experimental protocol. the procedure is color coded as follows: orange (online questionnaires) and gray (lab)

Eligible participants were first invited to complete an online questionnaire with their demographic details (e.g., sex, age) and the pre-session IRI and BFI. A laboratory session is then scheduled within two weeks of the completion of the online questionnaire, with a within-subjects design implemented. With fNIRS recording, all participants go through an initial 2-minute baseline condition where they are instructed to remain silent and not interact with each other. Then, in a counterbalanced fashion, participants go through the Naturalistic Conversation (i.e., participants act as themselves), Role-Play (i.e., participants act as other mutually known friends, classmates, or colleagues) and Role Reversal (i.e., participants act as each other) conditions. Each of these conditions last 5 min and present the same scenario. During these conditions, participants are instructed to interact freely with each other, albeit remaining seated at a fixed angle of approximately 45 degrees to each other. Participants spoke in their first languages (i.e., English for Singapore cohort and Italian for Italy cohort). At the end of the session, participants complete the post-session IRI. The procedure is summarized in Fig. 2.

### Data analysis

#### Pre-processing

**Fig. 3:**
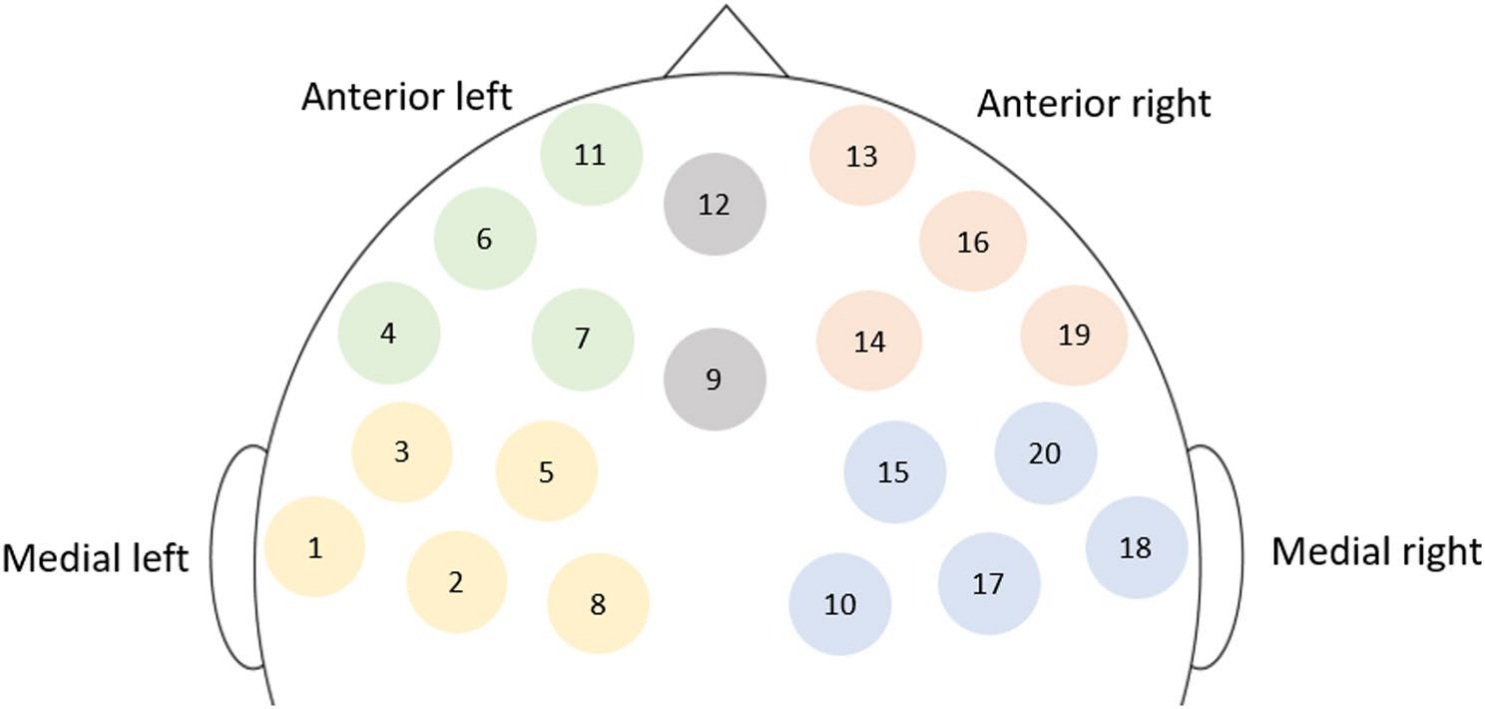
Schematic of anatomical clustering in the prefrontal cortex *Note*: Clusters are color coded: anterior left (green); anterior right (orange); medial left (yellow); medial right (blue). In this schematic, channels 9 and 12 are not used (gray)

Due to structural differences in conversations [88], the first and last minutes of the fNIRS recordings for Naturalistic Conversation, Role-Play and Role Reversal conditions are first truncated, preserving only the second, third and fourth minutes. Following that, all fNIRS files are pre-processed using *pyphysio* [89], where signal quality was assessed using machine learning [90]. Channels with bad signal quality are discarded. Motion artifacts are removed using a two-stage process [91] involving spline interpolation [92] and wavelet filtering [93] strategies. Following these corrections, the fNIRS signals are converted into concentration of oxygenated and deoxygenated hemoglobin (HbO and Hb) based on the Beer-Lambert law. In this study, only HbO data are used. In the filtering step, a third order, butterworth bandpass (0.01–0.5 Hz) Infinite Impulse Response bandpass filter is used [94] to exclude physiological and other noise. Finally, fNIRS signals are further aggregated into clusters, representing left anterior and medial, as well as right anterior and medial, anatomical regions of the prefrontal cortex. This aggregation is done by calculating the average HbO values based on channels that contributed to each cluster, only for clusters where the individual has two or more channels with good quality data [95]. The schematic summarizing how various fNIRS channels are clustered is seen in Fig. 3.

Time series HbO data were then obtained for each participant, for each cluster during each condition, which are used to calculate interpersonal brain-to-brain synchrony. Brain-to-brain synchrony for each homologous cluster, for each condition, between dyads is then calculated using Wavelet Transform Coherence across the frequencies from 0.01 to 0.20 Hz in steps of 0.01 Hz [96], which returns a value between 0 (no coherence or synchrony) to 1 (perfect coherence) based on the correlation of two signals across time and frequency (see Chang and Glover [97] for details on Wavelet Transform Coherence computation when applied to functional brain activity). This frequency range avoids the physiological noise occurring at greater than 0.2 Hz (e.g., breathing rate at ∼0.25 Hz and heart rate at ∼1.3 Hz) while including typical neuronal frequencies at ~ 0.025 Hz [94]. To ensure that synchrony occurred above chance levels, brain-to-brain synchrony values of individual participants from different dyads were also calculated (surrogate dyads), matching only based on the condition and brain region. This facilitates the comparison of synchrony values between true (i.e., participants who had gone through the conditions with each other) and surrogate (i.e., participants who had gone through the conditions with other people) dyads subsequently during preliminary data analysis. Brain-to-brain synchrony between surrogate dyads was measured similarly using Wavelet Transform Coherence.

To create a dyadic value for questionnaire data, raw scores from each member of the dyad are totalled.

#### Statistical analysis

Statistical analyses are performed on RStudio (v. 1.3.1093, Windows 64-bit). Firstly, following Reindl and colleagues [98], cluster synchrony values for surrogate and true dyads are compared. Only clusters where true dyads demonstrated significantly greater synchrony (one-tailed Mann-Whitney U test) are progressed for further analysis. Secondly, descriptive statistics for BFI and IRI are calculated. Cohort differences in personality and empathy are analyzed using two-tailed Mann-Whitney U test, as well as pre-post session changes in empathy using Wilcoxon Ranked Sum test.

To answer the first research question on the effect of condition, dyad sex and cohort on synchrony, a linear regression model is used. As for the second research question on personality, exploratory forward stepwise linear regression is employed. To answer the third research question on how these factors, together with personality variables, contribute to pre-post session changes in empathy, forward stepwise linear regression models are employed for each IRI subscale, as well as global IRI scores. In the stepwise regression models, potential predictors are inserted based on p-value and the stopping rule uses the model’s Akaike information criterion (AIC).

For all statistical analyses with multiple comparisons, Bonferroni correction is applied.

## Results

### Preliminary analysis

Comparative analyses between true and surrogate dyads across both cohorts revealed significantly greater brain-to-brain synchrony among true dyads for the medial left anatomical cluster (*U* = 29,186, *p* = 0.002) as well as overall fNIRS signals (Table 3). Therefore, further analysis only proceeded for the medial left cluster. Following aggregate analyses, the subsequent results presented only comprise dyads with valid fNIRS signals in the medial left cluster. Additionally, data from female-male dyads in the Singapore cohort were removed due to a lack of equivalent participants in the Italy sample.

**Table 3:** One-tailed Mann-Whitney U-test of synchrony between true and surrogate dyads

Cluster	True			Surrogate			U	*p*	95% CI
	*N*	Mean	SD	*N*	Mean	SD			
Anterior Left	297	0.472	0.044	293	0.465	0.043	46,864	0.052	[3.25e-05,1)
Anterior Right	250	0.483	0.041	243	0.476	0.045	33,722	0.017	[0.003,1)
Medial Left	221	0.480	0.042	228	0.469	0.042	29,186	0.002**	[0.005,1)
Medial Right	233	0.474	0.044	233	0.472	0.042	28,079	0.521	[0.003,1)
All	1001	0.477	0.043	997	0.470	0.043	546,371	1.69e-04***	[0.005,1)

*Note* Bonferroni correction applied over 4 clusters (*p* < 0.0125). The upper bound for the 95% confidence interval has been modified from infinity to 1 to aid interpretation, as the maximum theoretical value of synchrony according to the present analytic methods is 1

Descriptive statistics in dyadic personality traits between Singaporean and Italian cohorts are reported in Table 4. Additionally, comparative analyses revealed significant differences in Openness to Experience (U = 250.5, *p* = 0.01), Agreeableness (U = 650.5, *p* = 0.002) and Neuroticism (U = 284.5, *p* = 0.02) between cohorts. Specifically, Singaporean dyads tended to display lower openness to experience and neuroticism, but higher agreeableness than Italian dyads.

**Table 4:** Two-tailed Mann-Whitney U-test of personality between Singapore and Italy cohorts

Trait	Singapore			Italy			U	*p*	95% CI
	Mean	SD	Range	Mean	SD	Range			
Openness	67.62	7.93	51,88	72.57	6.54	54,85	250.5	0.01**	-9,-2
Conscientiousness	59	7.63	48,78	59.24	7.61	44,74	409.5	0.65	-5,3
Extraversion	49.67	10.26	34,73	50.38	7.37	31,63	417	0.73	-6,4
Agreeableness	66.62	7.67	54,80	60.02	5.42	49,71	650.5	0.002**	2,10
Neuroticism	47.71	9.34	24,64	52.55	6	41,66	284.5	0.02*	-9,-1

Descriptive statistics in pre- and post-session dyadic empathy between Singaporean and Italian cohorts are reported in Table 5. Comparative analyses between pre- and post-session empathy scores for each cohort are reported in Table 6, while the comparison of extent of change in empathy across cohorts are reported in Table 7. Results indicate that only the Singaporean cohort experienced significant increase in empathy (W = 124, *p* = 0.004), particularly in the empathic concern subscale (W = 9, *p* = 0.0009), whereas there were no significant changes in the Italian cohort. When comparing the extent and direction of change, it appears that there are significant differences in overall empathy (U = 533.5, *p* = 0.003), empathic concern (U = 566, *p* = 0.0005), as well as fantasy subscales (U = 515.5, *p* = 0.008) across cohorts. While the shifts in empathy tended to be positive after the session, it appears that the mean fantasy score decreased only in the Italian cohort.

**Table 5:** Descriptive statistics for dyad empathy by cohort

IRI	Singapore (Pre-)			Singapore (Post-)			Italy (Pre-)			Italy (Post-)		
	Mean	SD	Range	Mean	SD	Range	Mean	SD	Range	Mean	SD	Range
Overall	131.76	15.39	97,161	147.35	18.81	103,178	139.23	15.92	104,167	140.62	18.11	88.11,177.11
Fantasy	32.41	8.73	11,48	34.65	7.36	18,47	35.77	7.65	19,48	34.43	8.30	17,51
Empathic Concern	34.59	6.8	25,46	43.47	6.32	31,54	40.58	5.46	26,50	41.29	6.14	28,52
Perspective Taking	37.47	5.03	29,48	41.94	5.66	30,50	40.31	5.97	29,54	41.65	7.36	24,55
Personal Distress	27.29	7.18	10,40	27.29	8.79	12,38	22.57	7.81	5.15,38	23.25	6.80	11.66,37

**Table 6:** Pre- and post-session Wilcoxon rank-sum tests for dyad empathy by cohort

IRI	Singapore				Italy			
	Median Shift	W	95% CI	*p*	Median Shift	W	95% CI	*p*
Overall	16	124	5.5,28	0.004**	1	505.5	-1.84,4	0.50
Fantasy	2.5	116.5	-0.0,5	0.06	-1.5	269.5	-2,0	0.06
Empathic Concern	9	132.5	4.5,14	0.0009***	1	433.5	-0.83,2.5	0.22
Perspective Taking	4.5	125.5	1,8	0.02	1.96	571	0.01,3.01	0.03
Personal Distress	-0.5	58.5	-3.5,3.5	0.95	1	501	-1.2	0.36

*Note* Bonferroni correction applied over 4 subscales (*p* < 0.0125)

**Table 7:** Two-tailed Mann-Whitney U-test comparing dyad empathy change across cohorts

IRI	Singapore			Italy			U	*p*	95% CI
	Mean	SD	Range	Mean	SD	Range			
Overall	15.59	18.77	-12,51	1.39	9.28	-15.89,22	533.5	0.003**	3,20
Fantasy	2.24	4.51	-7,8	-1.35	4.27	-10,7	515.5	0.008**	1,6
Empathic Concern	8.88	8.38	-2,24	0.71	4.77	-12,11	566	0.0005***	3,11
Perspective Taking	4.47	6.67	-6.18	1.35	4.26	-8,8	466.5	0.07	0,6
Personal Distress	0	6.02	-10,9	0.68	4.56	-10,10	330	0.66	-3,2

*Note* Bonferroni correction applied over 4 subscales (*p* < 0.0125)

### Condition, sex and cohort effects on brain-to-brain synchrony

**Fig. 4:**
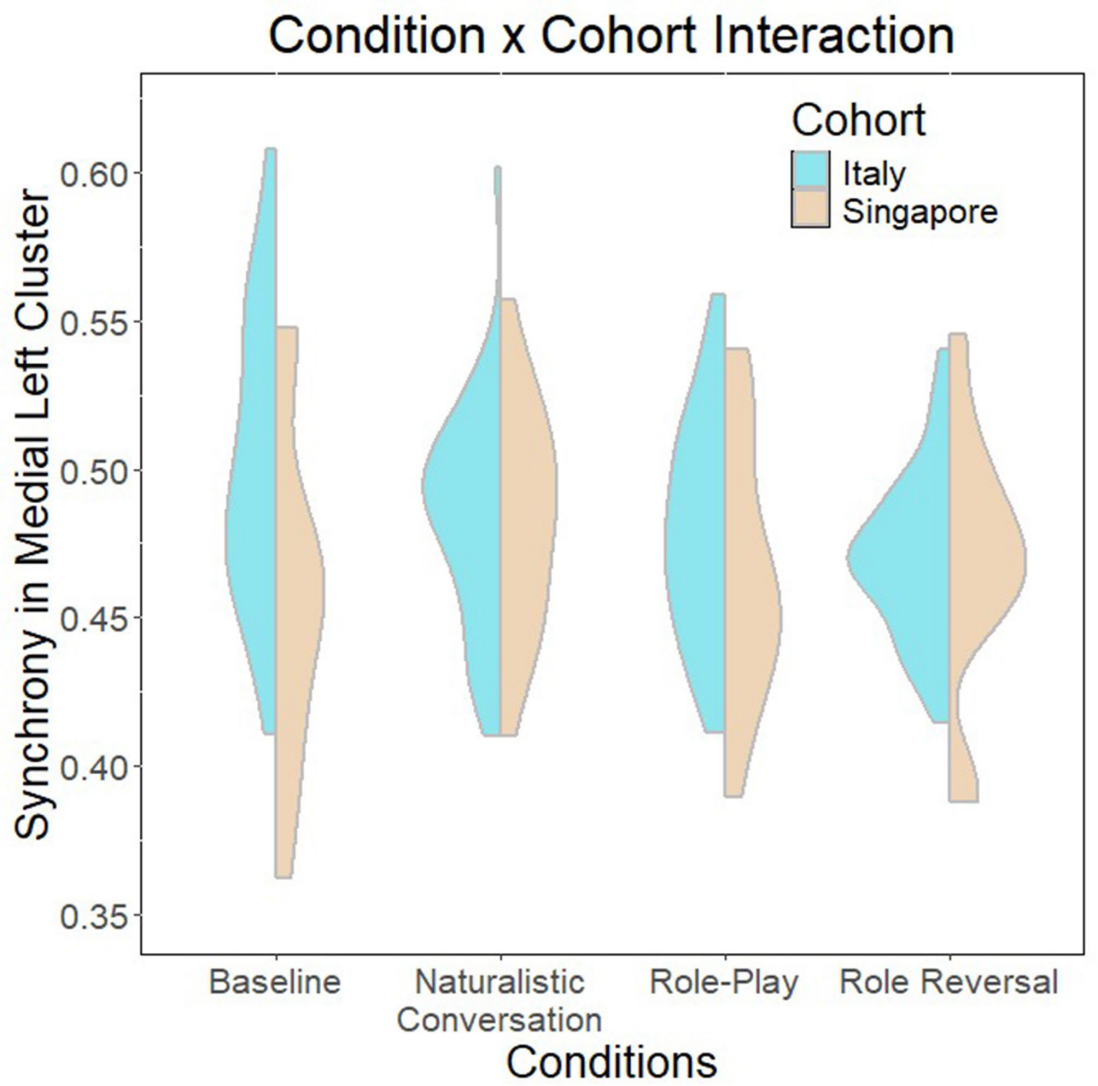
Two-way interaction between cohort and condition

**Fig. 5:**
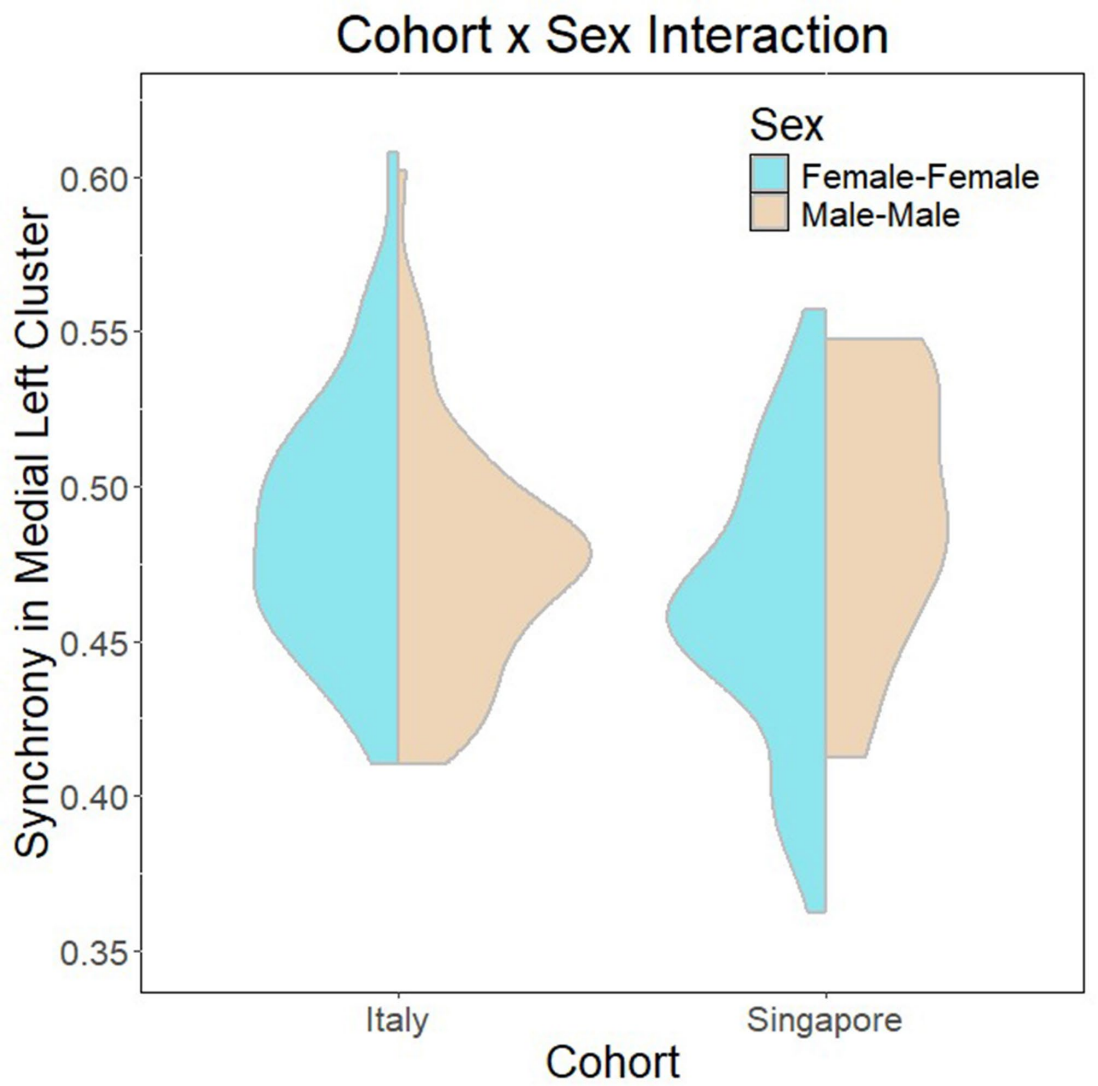
Two-way interaction between cohort and sex

**Fig. 6:**
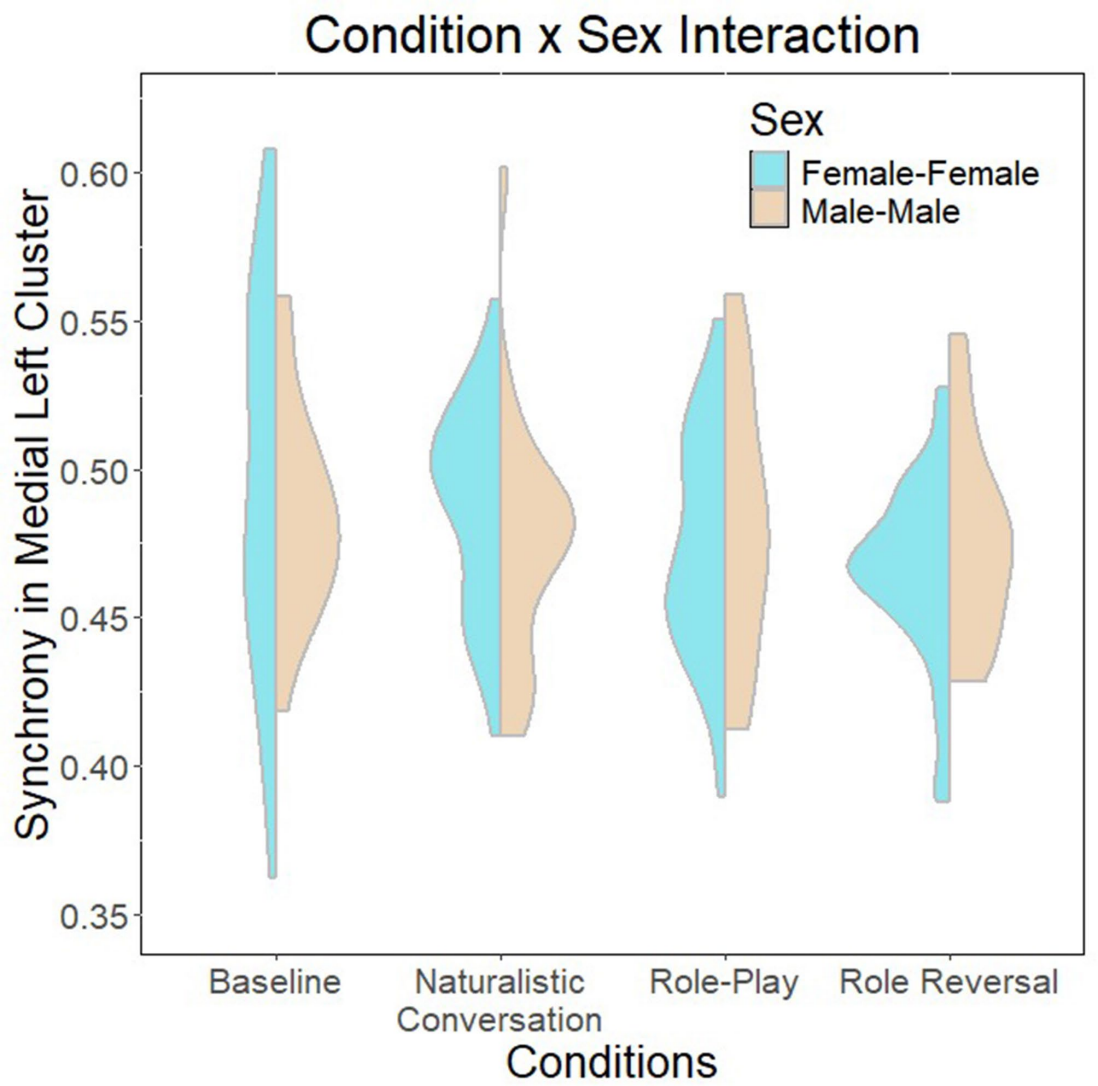
Two-way interaction between sex and condition

**Fig. 7:**
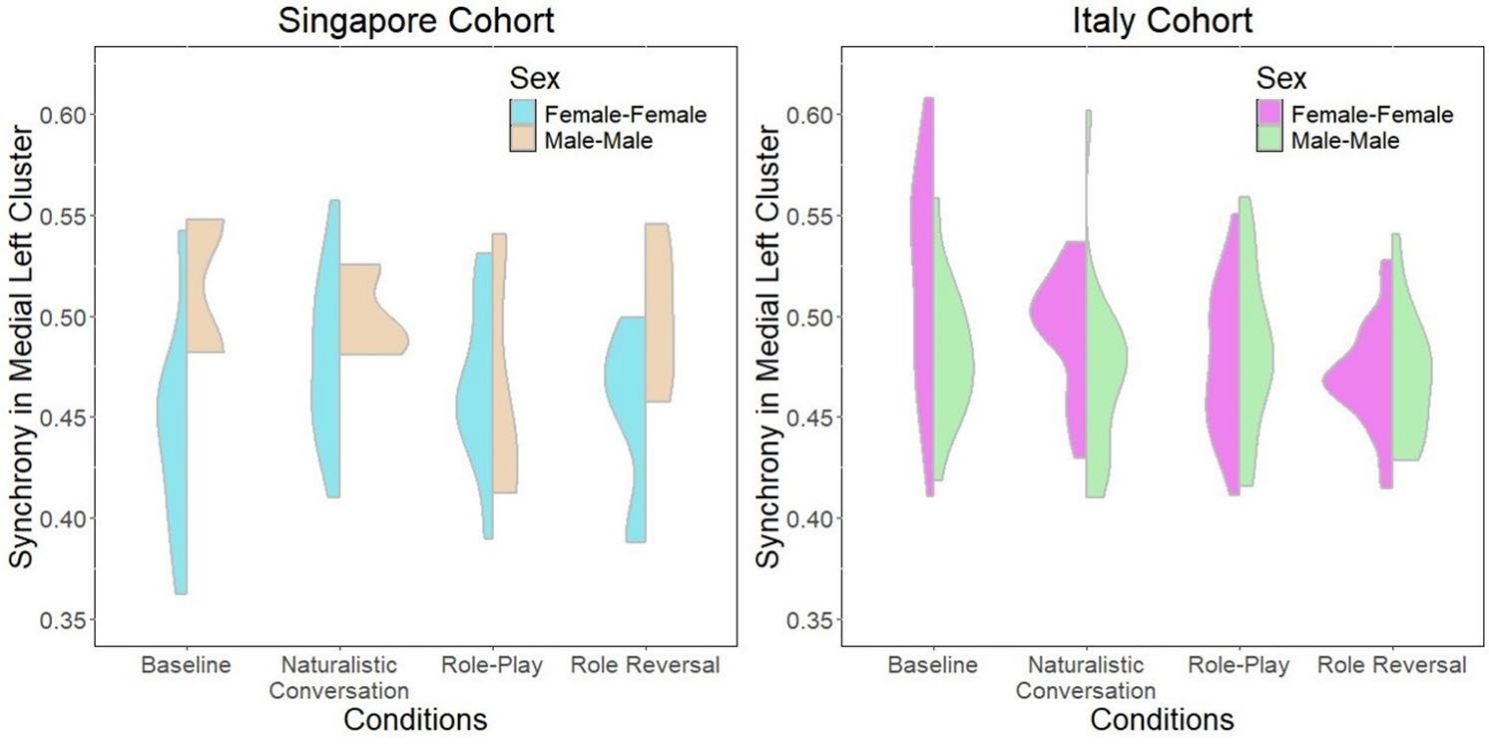
Three-way interaction between cohort, sex and condition. (Left) Data from Singapore cohort. (Right) Data from Italy cohort

The linear regression model to test Research Question 1 is summarized in Table 8. The overall model was significant (*F*(15,193) = 2.712, *p* = 8.63e-04), explaining up to 11% of the variance. There are significant main effects of cohort, condition and sex, as well as two-way interaction effects between condition and cohort, sex and cohort, as well as sex and condition (summarized in Figs. 4, 5 and 6). Finally, there are also significant three-way interactions between cohort, condition and sex (Fig. 7).

**Table 8:** Regression model for synchrony in medial left cluster by condition, cohort, and sex

Predictor	Beta	Std. Error	t	*p*	95% CI
Cohort (SG)	-0.07	0.02	-4.52	1.07e-05***	[-0.11,-0.04]
Condition (NC)	-0.02	0.01	-2.07	0.04*	[-0.05,-0.001]
Condition (RP)	-0.04	0.01	-3.41	7.88e-04***	[-0.06,-0.02]
Condition (RR)	-0.05	0.01	-4.02	8.25e-05***	[-0.07,-0.02]
Sex (M)	-0.04	0.01	-2.69	7.78e-03***	[-0.06,-0.01]
Cohort (SG) * Condition (NC)	0.06	0.02	2.83	5.14e-03***	[0.02,0.10]
Cohort (SG) * Condition (RP)	0.06	0.02	2.85	4.91e-03***	[0.02,0.11]
Cohort (SG) * Condition (RR)	0.06	0.02	2.47	0.01*	[0.01,0.10]
Cohort (SG) * Sex (M)	0.11	0.03	3.17	1.75e-03***	[0.04,0.17]
Condition (NC) * Sex (M)	0.01	0.02	0.79	0.43	[-0.02,0.05]
Condition (RP) * Sex (M)	0.04	0.02	2.30	0.02*	[0.01,0.08]
Condition (RR) * Sex (M)	0.04	0.02	1.98	0.05*	[0.0002,0.07]
Cohort (SG) * Condition (NC) * Sex (M)	-0.07	0.04	-1.48	0.14	[-0.15,0.02]
Cohort (SG) * Condition (RP) * Sex (M)	-0.12	0.04	-2.60	0.01*	[-0.20,-0.03]
Cohort (SG) * Condition (RR) * Sex (M)	-0.06	0.04	-1.39	0.17	[-0.15,0.03]
				F(15,193) = 2.71, *p* = 8.63e-04 Adj. R^2^ = 0.11	

*Note* Cohort was dummy coded with Italy cohort (IT): 0. Condition was dummy coded with Baseline: 0. Sex was dummy coded with Female: 0

### Personality analysis

Forward stepwise regression analysis revealed contrasting results by cohort. Tables 9 and 10 present generated final models of predictors of medial left cluster synchrony for the Singaporean and Italian cohort respectively.

Only one significant predictor of medial left cluster synchrony surfaced for the Singaporean cohort (male-male dyads positively predicts brain-to-brain synchrony in comparison with female-female dyads; β = 0.03, S.E. = 0.01, *p* = 0.04). The overall model explained 7% of overall variance (F(1,48) = 4.43, *p* = 0.04).

**Table 9:** Predictors of medial left cluster synchrony for Singaporean cohort

Predictor	Beta	Std. Error	t	*p*	95% CI
Sex (Male)	0.03	0.01	2.10	0.04*	[0.001,0.06]
				F(1,48) = 4.428, *p* = 0.04062 Adj. R^2^ = 0.07	

*Note* Dyad sex was dummy coded with female-female: 0

On the other hand, significant predictors of medial left cluster synchrony for the Italian cohort include condition (all experimental conditions negatively predict brain-to-brain synchrony in comparison with baseline; β(NC) = -0.02, S.E. = 0.01, *p* = 0.03; β(RP) = -0.02, S.E. = 0.01, *p* = 8.17e-03; β(RR) = -0.03, S.E. = 0.01, *p* = 2.19e-04), personality (specifically extraversion and openness) and sex (male-male dyads negatively predict brain-to-brain synchrony in comparison with female-female dyads; β = 0.04, S.E. = 0.02, *p* = 0.02). Particularly, extraversion (β = -0.001, S.E. = 0.0004, *p* = 2.94e-03) negatively predicts brain-to-brain synchrony, while openness to experience positively predicts brain-to-brain synchrony (β = 0.001, S.E. = 0.0005, *p* = 0.05). The overall model explained 14.2% of overall variance (*F*(7,151) = 4.74, *p* = 7.82e-05).

**Table 10:** Predictors of medial left cluster synchrony for Italian cohort

Predictor	Beta	Std. Error	t	*p*	95% CI
Condition (NC)	-0.02	0.01	-2.17	0.03*	[-0.04,-0.002]
Condition (RP)	-0.02	0.01	-2.68	8.17e-03**	[-0.04,-0.006]
Condition (RR)	-0.03	0.01	-3.79	2.19e-04***	[-0.05,-0.02]
Personality (Extraversion)	-0.001	0.0004	-3.02	2.94e-03**	[-0.002,-0.0004]
Sex (Male)	-0.02	0.01	-2.64	9.15e-03**	[-0.03,-0.004]
Personality (Openness)	0.001	0.0005	2.01	0.05*	[1.73e-05,0.002]
Personality (Conscientiousness)	-0.0006	0.0004	-1.51	0.13	[-0.001,0.0002]
				F(6,23) = 3.057, *p* = 0.02386 Adj. R^2^ = 0.2985	

*Note* Condition was dummy coded with Baseline: 0. Dyad sex was dummy coded with female-female: 0

### Empathy analysis

Tables 11, 12, 13, 14 and 15 summarize the final generated models of predictors of dyadic empathic change based on overall IRI scores, as well as for each subscale.

Only one significant predictor surfaced for overall empathic change (Singaporean cohort positively predicts increase in empathy after session; β = 10.67, S.E. = 3.78, *p* = 0.05). The overall model explained 27% of overall variance (*F*(3,55) = 8.14, *p* = 1.41e-04).

**Table 11:** Predictors of overall dyadic empathy change

Predictor	Beta	Std. Error	t	*p*	95% CI
Cohort (SG)	10.67	3.78	2.83	0.05*	[2.30,85.28]
Sex (Male)	-6.21	3.41	-1.82	0.07	[-12.26,3.95]
Personality (Openness)	-0.39	0.24	-1.66	0.1	[-1.14,-0.04]
				F(3,55) = 8.143, *p* = 1.409e-04 Adj. R^2^ = 0.27	

*Note* Cohort was dummy coded with IT: 0

Only one significant predictor surfaced for fantasy change (openness to experience negatively predicts increase in fantasy after session; β = -0.19, S.E. = 0.08, *p* = 0.02). The overall model explained 21.6% of overall variance (*F*(3,55) = 6.34, *p* = 9.07e-04).

**Table 12:** Predictors of dyadic fantasy change

Predictor	Beta	Std. Error	t	*p*	95% CI
Personality (Openness)	-0.19	0.08	-2.50	0.02*	[-0.43,-0.08]
Cohort (SG)	2.38	1.27	1.88	0.07	[-1.80,4.50]
Personality (Conscientiousness)	0.12	0.07	1.72	0.09	[-0.08,0.26]
				F(3,55) = 6.337, *p* = 9.073e-04 Adj. R^2^ = 0.2163	

*Note* Cohort was dummy coded with IT: 0

Changes in empathic concern were significantly predicted by cohort (Singaporean cohort positively predicts increase in empathic concern after session; β = 7.54, S.E. = 1.63, *p* = 2.4e-05) and sex (male-male dyads negatively predict increase in empathic concern after session; β = -3.75, S. E. = 1.57, *p* = 0.02). The overall model explained 36.3% of the overall variance (*F*(3,55) = 12.02, *p* = 3.86e-06).

**Table 13:** Predictors of dyadic empathic concern change

Predictor	Beta	Std. Error	t	*p*	95% CI
Cohort (SG)	7.54	1.63	4.61	2.4e-05***	[4.10,13.05]
Sex (Male)	-3.75	1.57	-2.39	0.02*	[-6.84,0.90]
Medial Left synchrony during NC	35.31	20.29	1.74	0.09	[-15.54,80.85]
				F(3,55) = 12.02, *p* = 3.864e-06 Adj. R^2^ = 0.3631	

*Note* Cohort was dummy coded with IT: 0. Dyad sex was dummy coded with female-female: 0

Only one significant predictor surfaced for perspective taking change (male-male dyads negatively predict increase in perspective taking after session; β = -3.06, S.E. = 1.30, *p* = 0.02). The overall model explained 21.9% of overall variance (*F*(3,55) = 6.42, *p* = 8.33e-04).

**Table 14:** Predictors of dyadic perspective taking change

Predictor	Beta	Std. Error	t	*p*	95% CI
Sex (Male)	-3.06	1.30	-2.34	0.02*	[-6.34,0.25]
Personality (Openness)	-0.16	0.08	-1.93	0.06	[-0.40,0.04]
Medial Left synchrony during RR	-37.58	20.45	-1.84	0.07	[-95.96,2.59]
				F(3,55) = 6.418, *p* = 8.328e-04 Adj. R^2^ = 0.2189	

*Note* Dyad sex was dummy coded with female-female: 0

Only one significant predictor surfaced for personal distress change (conscientiousness negatively predicts increase in personal distress after session; β = -0.18, S.E. = 0.090, *p* = 0.04). The overall model explained 5.53% of overall variance (*F*(1,57) = 4.40, *p* = 0.04).

**Table 15:** Predictors of dyadic personal distress change

Predictor	Beta	Std. Error	t	*p*	95% CI
Personality (Conscientiousness)	-0.18	0.09	-2.10	0.04*	[-0.36,0.06]
				F(1,57) = 4.397, *p* = 0.04046 Adj. R^2^ = 0.05532	

## Discussion

The present study set out with three research questions: firstly, to uncover culture and sex effects on interpersonal synchrony across varying social contexts, specifically role-play; secondly, to explore if personality factors contribute to interpersonal synchrony across cohorts; thirdly, to uncover predictors of pre-post empathy changes across cohorts.

Preliminary results indicated significantly greater brain-to-brain synchrony in the medial left cluster, which covers the left lateralization of the dorsolateral prefrontal cortex, Broca’s area and frontal eye fields, among true dyads as compared to surrogate data. Greater synchrony among true dyads alludes to unique interpersonal synchrony that arises as a result of the interaction in real-time. In the identified medial left cluster, with implicated functions in mentalization [71, 73], findings support the theorized relationship between mentalization and observed interpersonal synchrony [99, 100], potentially also recruiting mirror neurons found to be involved in the processing and imitation of expressive gestures [101, 102]. Additionally, other large functions associated with this cluster lie in language and speech processing [103–107], as well as working and episodic memory [108–110], involving unique social and particularly verbal cues as the conversations between participating dyads unfold, potentially explaining why synchrony is observed at higher levels in this cluster among true dyads. However, further studies are needed to map the contents of the dyad’s interaction to specific brain activity in this cluster to confirm this conjecture.

The regression model investigating culture, sex and condition effects on interpersonal synchrony to address research question 1 revealed significant main and interaction effects of all three factors. Notably, it appears that role-playing conditions are negatively associated with interpersonal synchrony. The findings largely concur with current literature that role-playing activities are characterized by a deactivation of self-related networks [64–66], perhaps implying a greater need for internal self-regulation as the individual inhibits typical social scripts used by themselves in favor of recreating behaviors that are associated with a different persona. This suggests a stronger need for internal self-regulation rather than interpersonal co-regulation and synchrony. A related study by Galbusera and colleagues [111] similarly demonstrates that greater interpersonal synchrony compromises self-regulation. However, when considering the effect of dyad sex in the condition by sex two-way interaction (Fig. 4), it appears that male-male dyads have greater synchrony during role-play conditions, while female-female dyads demonstrate greater synchrony in baseline and natural conversations rather than during role-play. While the current findings largely concur with existing literature conducted in naturalistic settings that females tend to demonstrate higher interpersonal synchrony [17, 18], the interaction uncovered here suggests that there are sex-related differences in the cognitive processing of different social contexts that result in different extents of synchrony observed. A possible explanation for this may be role-playing strategies adopted by different sexes. For example, females might tend to rely on internal modifications of social scripts to act out a role and therefore reduce interpersonal synchrony, while males might tend to rely on external cues from their interaction partner to modify their behavior during role-play, resulting in greater interpersonal synchrony. Of course, subsequent verification will be needed to confirm this theory.

The next research question is concerned with personality predictors of synchrony across cohorts. Based on the results of the forward stepwise regression, only synchrony in the Italian cohort could be predicted by some personality factors. Specifically, extraversion negatively predicted synchrony, while openness to experience positively predicted synchrony. It should be noted that these personality factors were representative of the participants’ ‘true’ selves, rather than the personalities of personas chosen during role-play conditions, which were not measured in the present study. As previously uncovered in Lim and colleagues [49] and Jeng and Teng [48], openness to experience is a significant predictor of role-playing experience, and may serve as a positive contributor to more immersive behaviors during these interactions [112]. In the context of interpersonal synchrony, greater openness may allow individuals to be more receptive to their partners’ cues during a social interaction and enable greater co-regulation. On the other hand, while the finding on extraversion aligns with Tschacher and colleagues [113], it contrasts with that of Arellano-Véliz and colleagues [45]. A possible explanation put forth by Tschacher and colleagues [113] lies in intentionality; extraverted participants may prioritize socializing as compared to attending to the task. In our context, this may manifest in role-breaking behaviors and lower immersion in the interaction tasks, resulting in lower synchrony overall, but would require further studies to verify this conjecture.

The third research question focuses on the impact of culture, sex, and personality variables on observed changes in dyadic empathy across the session. In alignment with our comparative results, culture was consistently brought up as significant contributors of empathy change, particularly in the overall and empathic concern measures where significant cross-cohort differences were detected. The observed trend may have to do with larger transient fluctuations in empathy among Singaporeans as compared to Italians. In general, pre-session empathy scores tended to be lower in Singapore as compared to Italy cohorts, and self-reported empathy tended to experience greater increases after the session as compared to the Italy cohort. This may suggest a greater likelihood for Singaporeans to experience context-dependent changes in empathy, rather than express more stable, trait-dependent empathy across a variety of contexts. Lower baseline empathy is corroborated by other findings which found lower trait empathy levels among participants of Eastern rather than Western cultures [55, 56], particularly in empathic concern [57]. In the same series of studies, greater changes in state (i.e., transient) empathy among Asian participants were also found for negatively valenced and neutral stimuli [55]. Therefore, it may be premature to conclude that role-playing activities are less effective in promoting empathy for Italian participants as compared to Singaporean participants. Further studies would be required to implement and evaluate culture-sensitive role-playing interventions and their effect on empathy, while making use of multiple measures of empathy (e.g., third-party ratings, behavioral expressions of empathy) to confirm the pattern of findings.

### Limitations and future studies

Limitations in the interpretation of present findings and suggestions of further studies have been raised throughout the discussion above. Here, other limitations concerning the research design are delineated. Firstly, high interpersonal synchrony is not always indicative of positive outcomes; synchrony during moments of negative affect or conflict may result in lower relationship quality and poorer coping [114–117]. When considered in the context of the present study, an absence of brain-to-brain synchrony as a significant contributor of empathy change may not imply that interpersonal synchrony has no relationship to empathy changes. Rather, it may be more crucial to consider if synchrony fluctuates as a function of changes in the quality and contents of social interaction. This may be achieved in future studies by analyzing smaller event windows.

Secondly, the language used by participants across cohorts is different. This may have implications particularly in the identified medial left cluster of the prefrontal cortex, which is associated with language processing [103–107]. Due to existing language barriers between cohorts, it is prudent to default to the respective commonly spoken languages in a naturalistic research design for the present study. However, the study assumes that English and Italian are of first-language familiarity and fluency for Singaporean and Italian participants respectively. Future studies attempting cross-cultural comparisons may need to consider the language used and implement checks to ascertain participants’ language proficiency. The exclusion of male-female dyads due to a lack of an equivalent participant demographic in the Italian cohort is also a missed opportunity to further study sex effects in mixed dyads and may be explored in the future.

Thirdly, the study operationalizes interpersonal synchrony by using hyperscanning fNIRS to detect patterns of brain activation. fNIRS is technically limited to only imaging cortical areas of the brain, and this study further only focuses on the prefrontal cortex. In a review article, Levy and colleagues [118] highlight the integration of multiple modalities of synchrony, providing the example of combining behavioral with neural synchrony, to enhance ecological validity when implementing naturalistic designs. This hints at potential future studies that can exploit multimodal sources of information to arrive at a more nuanced picture of culture, sex and personality influences on social interactions and synchrony. In fact, studies are beginning to consider multiple measures of synchrony (e.g., behavioral and brain-to-brain synchrony in Chuang and Hsu [119]). Future work adopting the same paradigm as the present study may similarly consider behavioral output when measuring synchrony, such as in terms of eye gaze, or communicative gestures or body postures. Furthermore, due to differences in logistics and resources available, the NIRS hardware and accompanying software models employed during each wave of data collection are not identical. There may be device- or system-related differences in the manner the signals are recorded across cohorts. However, it should be noted that fNIRS studies have been found to be generally reproducible [120], as long as standardized processing pipelines are used to reduce differences found in raw signals. Additionally, the MNI coordinates reported in the present study are only theoretical projections as there was a lack of structural imaging in the protocol, as well as information regarding participants’ head diameters and NIRScap sizes used. To improve the precision of fNIRS imaging, and particularly to enhance the replicability of results, future studies may consider collecting these data to implement modeling or projective techniques that can translate optode positions consistently to cortical regions.

## Conclusions

Social interactions from which interpersonal synchrony arises are shaped by the background characteristics of participating individuals: from the culture from which they hail, to biological sex, as well as personality traits. In addition, the context of the social interaction is also a crucial external influence on synchrony. Using a novel role-play design where dyads either interact as themselves or others (another person not in the room, or their participating partner), the present study investigates the effect of experimental condition, culture, sex and personality on brain-to-brain synchrony, and attempts to connect these variables to changes in dyadic empathy from pre- to post-experimental session. Findings uncovered significant main and interaction effects of condition, culture and sex on synchrony, as well as differential effects of personality on synchrony across cohorts. Finally, culture was found to predict changes in dyadic empathy.

## Data Availability

The datasets used and/or analyzed during the current study are available from the corresponding author on reasonable request.

## Abbreviations

AIC: Akaike Information Criterion
BFI: Big Five Inventory
EEG: Electroencephalogram
fNIRS: functional Near-Infrared Spectroscopy
Hb: Deoxygenated Hemoglobin
HbO: Oxygenated Hemoglobin
IRI: Interpersonal Reactivity Index
IT: Italy Cohort
MNI: Montreal Neurological Institute
NC: Naturalistic Conversation Condition
RP: Role-Play condition
RR: Role Reversal condition
SG: Singapore Cohort
